# A rare case report of paratesticular spindle cell tumor: Inflammatory myofibroblastic tumor

**DOI:** 10.1016/j.ijscr.2023.108235

**Published:** 2023-04-25

**Authors:** Stefanus Purnomo, Andika Afriansyah, Hendy Mirza, Doddy Hami Seno, Nugroho Purnomo, Moammar Andar Roemare Siregar

**Affiliations:** a Department of Surgery, Division of Urology, Persahabatan General Hospital, Jakarta, Indonesia; b Department of Surgery, Division of Urology, Persahabatan General Hospital - Faculty of Medicine, Universitas Indonesia, Jakarta, Indonesia

**Keywords:** Spindle cell tumor, Paratesticular tumor, Inflammatory myofibroblastic tumor

## Abstract

**Introduction and importance:**

Spindle cell tumors in the paratesticular region are infrequent. Accurate diagnosis requires clinical assessment, pathological analysis, and immunohistochemical (IHC) analysis.

**Case presentation:**

The present study reports a 33-year-old male who presented with a painless mass in his right testis. The mass grew from the size of a marble to that of a tennis ball in two years. Physical examination and ultrasonography revealed a solid mass in the right paratesticular area. The patient underwent a right paratesticular tumor excision, without interfering the right testis. Histopathologic analysis showed spindle cell tumor appearance referring to inflammatory myofibroblastic tumor (IMT).

**Clinical discussion:**

IMT, also known as inflammatory pseudotumor, is a rare and benign neoplasm that can occur anywhere. Diagnosis is challenging, because it mimics other neoplasms. Immunohistochemical profiles were decisive in concluding a definitive diagnosis. Treatment, on the other hand, depends on tumor burden and connectivity to other region.

**Conclusion:**

Spindle cell tumors are very rare and can be treated with simple excision if no organ adhesion is detected. Therefore, right orchidectomy was not required in our case.

## Highlights

• A paratesticular spindle cell tumor leading to inflammatory myofibroblastic tumor is very rare worldwide.
• Pathological analysis alone is insufficient to gain a definitive diagnosis.
• Not all paratesticular spindle cell tumors needed orchidectomy; it depends on each case.
• Prognosis for this case is favorable, but do not underestimate it in the long term.

## 1 Introduction

Tumors originating from paratesticular region are categorized as rare tumors, with very few cases occurring worldwide. Paratesticular tumors refer to neoplasms located in the epididymis, spermatic cord, and their surrounding structures within the scrotal region. Although these tumors are very rare, a diverse range of paratesticular cysts and tumors may manifest as inguinal masses within the scrotum [1]. Paratesticular spindle cell tumors (PSCTs) are very rare benign tumors typically presenting as slowly growing masses. The precise diagnosis for these tumors can be challenging during both pre- and intraoperative stages [2]. Here we present a case of paratesticular spindle cell tumor with immunohistochemistry findings leading to inflammatory myofibroblastic tumor. The patient underwent tumor excision with testis-sparing surgery. This case report was constructed based on SCARE guideline [3].

## 2 Presentation of case

**Fig. 1:**
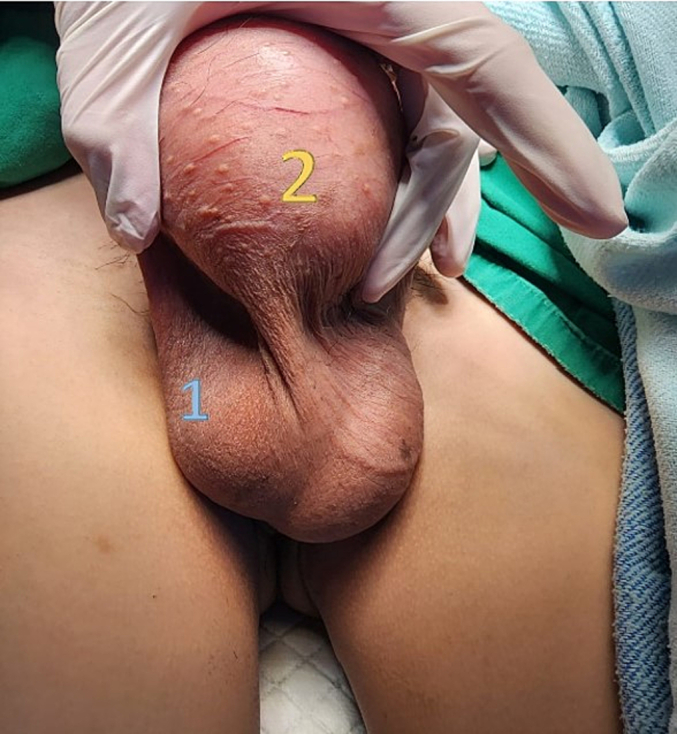
Physical examination identifying the right paratesticular tumor oval round shaped with a size equivalent to that of a tennis ball. 1: Right testis. 2: Tumor.

**Fig. 2:**
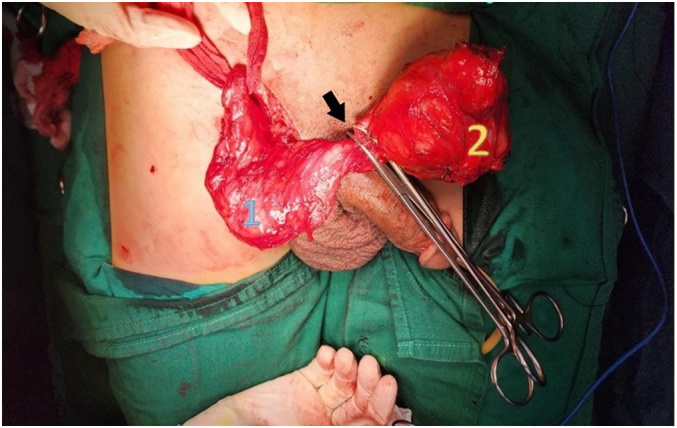
Intraoperative finding of the tumor originated from the parietal layer of tunica vaginalis with clear separation and no adhesions to other surrounding structures. The surgical clamp marked the base or origin of the tumor (arrow). 1: Right testis. 2: Tumor.

**Fig. 3:**
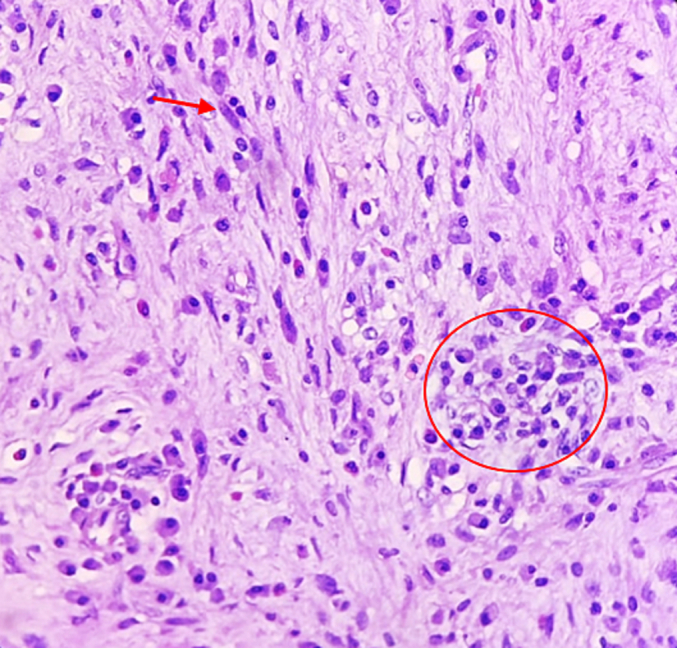
Tumor cells (arrow) arranged in short clustered and storiform between inflammatory cells (circle) and myxoid stroma showing thin circumference connective tissue, HE 400×.

**Fig. 4:**
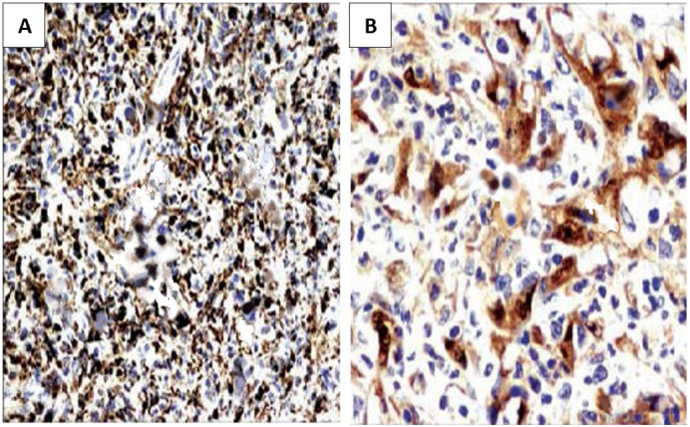
Immunohistochemichal stains for CD68 with 100× (A) and S100 protein with 400× (B) showing inflammatory myofibroblastic tumor characteristics.

A 33-year-old man came to the urology office presented with a painless mass in his right testis. Starting from a marble size mass to that of a tennis ball in the past two years. The patient had no history of tumors, familial tumors, or other systemic diseases, but had a smoking habit of consuming at least one box of cigarettes per day. Physical examination identified an oval round shaped mass without any adhesion to the testis (Fig. 1). There was no enlargement of lymphatic nodules regionally, and bilateral scrotum ultrasonography revealed a right testicular solid mass with size of 6.9 cm diameter pushing the ipsilateral epididymis. Routine laboratory checkup and other examinations (AFP, β-HCG & LDH) were within the normal range values. Pre-operative assessment was cleared and found nothing extraordinary. The patient underwent surgery in a supine position with spinal anesthesia. An experienced urologist performed a right inguinal incision and performed a regional assessment before excision. The respective spermatic cord was identified and while applying gentle traction in a cephalad direction and external pressure to the ipsilateral hemiscrotum, the testis and the tumor was gently pushed and both of them were delivered into the surgical field. We found that the tumor originated from the parietal layer of the tunica vaginalis. It was separated from the testis, which made it easy to remove, and no infiltration was observed in nearby structures such as the testis, epididymis, or the contents of the spermatic cord (Fig. 2). No adhesions and any other malignancies were found between the testis and the tumor. Therefore, testis-sparing surgery was done by complete excision without orchidectomy. Pathological analysis revealed the diagnosis of right paratesticular tumor. Macroscopically, the mass measured 8 × 8 × 7 cm, brownish colored, tender and smooth surface with thin capsule. Microscopic examination showed thin circumference connective tissue, composed of tumor cell proliferation with short clustered, round/oval core, spindle, light pleomorphic, smooth chromatin, and eosinophilic cytoplasm (Fig. 3). Lymphoplasmacytic inflammatory cells were spotted in certain clustered areas, forming lymphoid follicles with a germinativum centrum, while hyalinized stroma was also found in other clustered areas. The histological findings led to the conclusion of a spindle cell tumor, specifically an inflammatory myofibroblastic tumor. Immunohistochemistry showed strong expression from the spindle cells consisting of CD68, S100 protein, and vimentin, which brought out the prolongations of cytoplasm (Fig. 4). The patient was discharged after two days postoperative care without any complaints or complications and was prescribed oral cephalosporin antibiotics for seven days. We suggested that the patient undergo a contrast computed tomography (CT) scan two weeks postoperatively, which revealed no mass in the right scrotum and no metastasis to other regions. The patient reported no re-occurrence and no other symptoms felt until now. We encouraged that the patient undergo follow-up examinations one year and three years after the operation, including routine blood works and physical examination of the operation site and its surroundings. Based on our last follow-up on, nothing remarkable was reported.

## 3 Discussion

Intrascrotal tumors could be testicular or paratesticular, PSCTs are rarer (7–10 %) and only 30 % of them are malignant. PSCTs are heterogeneous group of tumors, ranging from benign lesions to malignant tumors. The most common malignant tumors in the paratesticular area are sarcomas, and among adult sarcoma cases, rhabdomyosarcomas account for approximately 24 % and only 7–10 % of them involve the paratesticular region [4], [5]. Due to their rarity, PSCTs pose diagnostic and therapeutic challenges for clinicians. PSCTs account for less than 5 % of all paratesticular tumors. PSCTs consist of spindle-shaped cells that resemble smooth muscle cells, fibroblasts, or myofibroblasts. The differential diagnosis includes leiomyoma, leiomyosarcoma, fibroma, fibrosarcoma, synovial sarcoma, lipoma, liposarcoma, and malignant fibrous histiocytoma. Between the malignant tumors, liposarcoma is the most common type, making up for 46.4 % of cases. Leiomyosarcoma is the next most common, accounting for 20 %, followed by malignant fibrous histiocytomas at 13 %, and embryonal rhabdomyosarcoma at 9 % [6]. IMT, also known as inflammatory pseudotumor, is a rare and benign neoplasm that can occur in a variety of organs including the lung, liver, gastrointestinal tract, bladder, and soft tissues [7]. Despite its benign nature, IMT can cause significant morbidity if left untreated, and can sometimes recur after resection. The exact cause of IMT is not fully understood, but it is believed to arise from the proliferation of myofibroblasts and inflammatory cells. IMT is often associated with a history of inflammation or trauma, but the specific triggering event is not always clear. Some cases of IMT have been linked to genetic abnormalities, including chromosomal rearrangements and mutations in certain genes, such as ALK and ROS1 [8].

The diagnosis is very challenging due to the rarity of these tumors and the lack of specific clinical features as their clinical presentation and imaging features are non-specific and can mimic other neoplasms or inflammatory conditions. Ultrasonography, CT scan, and magnetic resonance imaging (MRI) can be used for diagnosis and staging [9]. Fine needle aspiration cytology (FNAC) demonstrated by K D et al. showed good results for initial diagnosis of soft tissue spindle cell tumors [10]. Histopathologic examination of the biopsy specimen typically reveals a proliferation of spindle-shaped myofibroblasts, often admixed with inflammatory cells such as plasma cells, lymphocytes, and eosinophils. IHC staining is perfect in confirming the diagnosis and identifying specific molecular markers [11].

Treatment of these rare cases depends on the histological subtype, size, and stage of the tumor. The mainstay of treatment is surgical resection with negative margins. Tumor-sparing surgery was performed in our case as there was no discernible tissue connection to the testis. A different approach would be considered if our intraoperative findings revealed malignancy. Radical orchiectomy would be considered for such cases, followed by pathological analysis, chemotherapy and other treatments if necessary. If a tumor is difficult to remove due to its location, alternative therapies such as chemotherapy or radiation therapy may be utilized as an alternative [12]. Targeted therapies, specifically for patients with ALK and ROS1 rearrangements, show promises for treating IMT, but their effectiveness is still uncertain and requires further research [13]. Most patients have good prognosis after complete removal, however, varies depending on the tumor's characteristics [14]. Regular monitoring, including clinical assessments and surveillance imaging, is advised to evaluate recurrences or progression of the disease [15].

## 4 Conclusion

In conclusion, our case presents a rare and benign neoplasm that can occur in various organs. While the exact etiology is not fully understood, the proliferation of myofibroblasts and inflammatory cells is believed to play a role in its development. Diagnosis can be challenging, but pathological findings and IHC will be sufficient for definitive diagnosis. Surgical resection with close follow-up is the preferred treatment for all patients. Despite its generally favorable prognosis, some cases of could recur or metastasize, underscoring the importance of long-term monitoring for patients with this condition.

## Patient's consent

Written informed consent was obtained from the patient for publication of this case report and accompanying images. A copy of the written consent is available for review by the Editor-in-Chief of this journal on request.

## Ethical approval

Ethical approval was provided by the authors institution.

## Funding

There was no financial support associated with the publication of this article.

## Guarantor

Moammar Andar Roemare Siregar.

## CRediT authorship contribution statement

Study concept and design: MARS, AA, SP
Data collection: MARS, AA, SP
Analysis and interpretation of case presentation: All Authors
Drafting of the manuscript: MARS, NP, SP
Critical revision of the manuscript: AA, HM, DHS, MARS
Study supervision: HM, DHS.

## Conflict of interest

The authors declare no competing interests that may affect the publication of this article.
